# Xerophthalmia secondary to bowel malabsorption after Roux-en-Y gastric bypass

**DOI:** 10.3205/oc000233

**Published:** 2024-01-24

**Authors:** José Arturo Oyervides-Alvarado, Schenny Murra-Anton, Ethel Guinto-Arcos, Laura Alejandra González-Dibildox, Nallely Ramos-Betancourt

**Affiliations:** 1 Asociación para Evitar la Ceguera en México IAP, Hospital Dr. Luis Sánchez Bulnes, Mexico City, Mexico

**Keywords:** xerophthalmia, vitamin A deficiency, bowel malabsortion, Bitot’s spots, Lynch syndrome, case report

## Abstract

**Introduction:**

Vitamin A is a fat-soluble vitamin, obtained through diet. Vitamin A deficiency is the leading cause of preventable blindness in children in developing countries due to impaired intake (Phanachet et al. 2018). Nevertheless, it is uncommon in the developed world where malabsorption takes a prominent role.

**Case description:**

A fifty-one-year-old female presented complaining of foreign body sensation, pain, tearing, fluctuating visual acuity, nyctalopia, diarrhea, polyphagia and weight loss. She had history of Roux-en-Y gastro-jejunal bypass, Lynch syndrome and right hemicolectomy with ileo-colonic anastomosis, she also referred to an additional unspecified bowel resection. In the ophthalmologic examination, best corrected visual acuity was 20/30, intraocular pressure was 11 mmHg in both eyes. Anterior segment biomicroscopy revealed a dry and thickened conjunctiva with wrinkles, multiple grey-white small, round, confluent, foamy lesions in the interpalpebral conjunctiva of both eyes, compatible with Bitot’s spots, and superficial punctate keratitis.

**Discussion:**

The rise of bariatric surgery, inflammatory bowel disease and end stage liver disease has led to an increase in cases of malabsorption syndrome and nutrient deficiencies in the developed world. Retinoids are essential for corneal and conjunctival epithelial cells differentiation and its deficiency is associated with a wide spectrum of ocular surface manifestations known as xerophthalmia. In this case, a gastric bypass and another unspecified bowel resection should raise the suspicion of malabsorption and nutrient deficiencies. In our patient, the diagnosis was made early and appropriate treatment was implemented before irreversible damage arose, however, vitamin A deficiency can be easily overlooked.

**Conclusion:**

In patients with xerophthalmia, interrogation should include previous history of gastrointestinal surgery, especially since bariatric surgery has become a popular technique. This is, to our knowledge, the first case report of xerophthalmia in a patient with Lynch syndrome.

## Introduction

Vitamin A is a fat-soluble vitamin, obtained through diet, absorbed in the small intestine and stored in the liver in high amounts [1]. Worldwide, vitamin A deficiency (VAD) is the leading cause of preventable blindness in children in developing countries due to impaired intake [1]. Nevertheless, it is uncommon in the developed world where malabsorption takes a prominent role. Fat malabsorption can be related to gastrointestinal infections, inflammatory bowel diseases (IBD) or procedures like bariatric surgery. Gastric bypass, sleeve gastrectomy, and biliopancreatic diversion with duodenal switch are among the most common bariatric procedures performed. There are no specific recommendations regarding bariatric surgery in patients with Lynch syndrome [2]. We present a case of xerophthalmia secondary to multiple gastrointestinal resections.

## Case description

A fifty-one-year-old female presented complaining of 6 weeks of slowly increasing foreign body sensation, pain, tearing, fluctuating visual acuity and nyctalopia. She had a history of diarrhea, lasting for 2 months (6–10 depositions per day), polyphagia and weight loss of 15 kg in the last 3 months. Family history was positive for systemic hypertension, hereditary non-polyposis colorectal cancer (Lynch syndrome), ovary, colon and uterine cancer. Past medical history was positive for stage III obesity (2003), type 2 diabetes mellitus (2003), dyslipidemia (2003), Roux-en-Y gastro-jejunal bypass (2006), Lynch syndrome and colon adenocarcinoma (stage IIa) diagnosed in 2013 treated with a right hemicolectomy with ileo-colonic anastomosis. She also referred to an additional unspecified bowel resection (2015).

**Figure 1:**
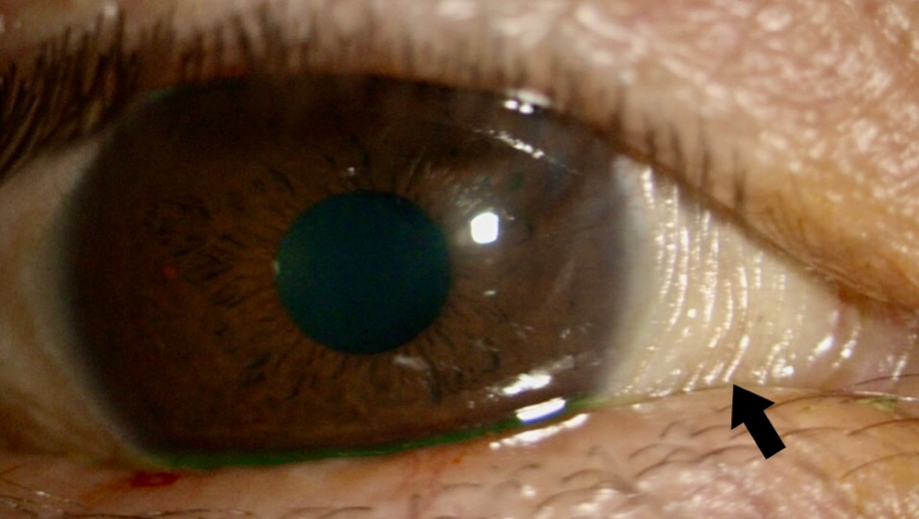
Thickened and dry conjunctiva with folds (black arrow)

**Figure 2:**
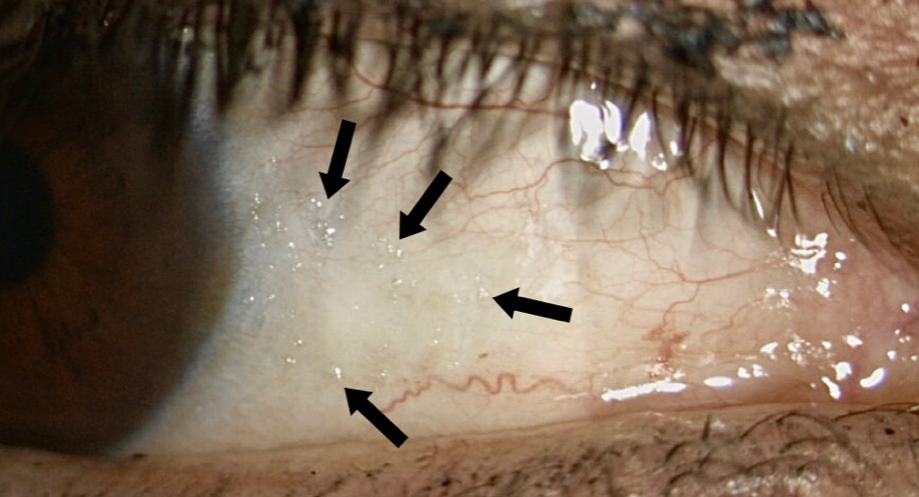
Bitot’s spots: grayish, small, foamy, round and confluent spots in the interpalpebral conjunctiva (black arrows)

In the ophthalmologic examination, best corrected visual acuity was 20/30 and intraocular pressure was 11 mmHg in both eyes. Anterior segment biomicroscopy revealed a dry and thickened conjunctiva with wrinkles (Figure 1 (Fig. 1)), multiple subtle grey-white small, round, confluent, foamy lesions in the interpalpebral conjunctiva of both eyes, compatible with Bitot’s spots (Figure 2 (Fig. 2)), and a superficial punctate keratitis in both eyes. The rest of the ophthalmologic examination was unremarkable in both eyes.

Xerophthalmia due to malabsorption was suspected. Topical preservative free lubricants and topical fluorometholone were prescribed. Interconsultation with gastroenterology and blood tests were requested.

On blood work, vitamin A was 0.1 mg/dl (normal range 0.4–1.2 mg/dl), vitamin D 5.1 mg/dl (30–80 mg/dl) and albumin 2.3 mg/dl (3.5–4.8 mf/dl). With these results the diagnosis of xerophthalmia stage X2 secondary to malabsorption syndrome was confirmed. Vitamin A 200,000 UI supplementation was added to treatment and the patient was referred to a third-level institution for systemic management and control. Six months later, a complete resolution of ocular and systemic signs and symptoms was achieved, including dry eye, nyctalopia and diarrhea.

## Discussion

VAD continues to be a major health problem in underdeveloped countries [1]. Nevertheless, the rise of bariatric surgery, IBD and end stage liver disease has led to an increase in cases of malabsorption syndrome and nutrient deficiencies in the developed world [1]. Vitamin A is stored in the liver in the form of retinyl palmitate and normal adult liver stores are sufficient to fulfil daily requirements for up to 2 years [3]. We believe that is why ocular symptoms presented after a few years of intestinal malabsorption in our patient.

Vitamin A plays an essential role in the phototransduction process of human vision [3]; in the form of 11-cis-retinal, it couples with the protein opsine to form rhodopsine. Although nyctalopia is usually the earliest symptom of VAD [3], our patient sought medical attention mainly because of dry eye symptoms.

Retinoids are essential for the corneal and conjunctival epithelial cells differentiation and its deficiency is associated with a wide spectrum of ocular surface manifestations known as xerophthalmia [1] (practically pathognomonic). Xerophthalmia produces tear film instability which leads to an ocular surface-related form of evaporative dry eye. The presence of early tear film breakup has been proposed to be the main basis for tear film hyperosmolarity, which leads to the release of inflammatory mediators and proteases causing goblet and epithelial cell loss, as well as epithelial glycocalyx damage [4]. Bitot’s spots are conjunctival patches of dry-looking epithelium with overlying foamy material mainly composed of keratin and a gas-forming bacteria (*Corynebacterium xerosis*). Conjunctiva might have a folded and dry appearance. Corneal xerosis can be observed as punctate superficial epithelial lesions and can progress to keratomalacia, which might advance to corneal thinning or even perforation. If the ocular manifestations are recognized early, they are reversible with sustained replacement therapy [3].

In this case, a gastrojejunal anastomosis (gastric bypass) and other unspecified bowel resection should raise the suspicion of malabsorption and nutrient deficiencies. After bariatric procedures, it has been described that VAD can develop by malabsorption related to the exclusion of gastrointestinal segments and a decrease in food intake [2]. Previous studies have reported VAD rates ranging from 60 to 70% after performing a biliopancreatic diversion, while rates of around 10% are reported after a Roux-en-Y gastric bypass [5]. After undergoing this type of procedure it is important to maintain strict nutritional vigilance.

In our patient, the diagnosis was made early and appropriate treatment was implemented before irreversible damage arose. However, VAD can be easily overlooked, especially in developed countries where cases of vitamin A deficiency are scarce. Patients who undergo bariatric surgery, and especially in the context of concomitant intestinal diseases, should stay closely monitored from a nutritional point of view, as well as regularly tested for vitamin deficiencies to avoid late complications. This case demonstrates the importance of a complete dietary history and identification of risk factors for VAD. There are no specific recommendations for patients with Lynch syndrome that contraindicate bariatric surgery. However, it is reasonable to recommend a procedure that causes minimum anatomic disruption like a gastric sleeve, and only after a complete preoperative study including an esophagogastroduodenoscopy.

## Conclusion

In patients with xerophthalmia, interrogation should include previous history of gastrointestinal surgery, especially since bariatric surgery has become a popular technique. Physicians, especially ophthalmologists, should be familiar with the etiology and subtle clinical manifestations of this disease as their timely diagnosis can completely resolve symptoms; however, an undetected chronic VAD might cause permanent visual impairment. This is, to our knowledge, the first case report of xerophthalmia in a patient with Lynch syndrome.

## Notes

### Competing interests

The authors declare that they have no competing interests.
